# Evaluation of Information in Summaries of Product Characteristics (SmPCs) on the Use of a Medicine in Patients With Hepatic Impairment

**DOI:** 10.3389/fphar.2019.01031

**Published:** 2019-09-17

**Authors:** Rianne A. Weersink, Lotte Timmermans, Margje H. Monster-Simons, Peter G. M. Mol, Herold J. Metselaar, Sander D. Borgsteede, Katja Taxis

**Affiliations:** ^1^Unit of Pharmacotherapy, Epidemiology & Economics, Department of Pharmacy, University of Groningen, Groningen, Netherlands; ^2^Department of Clinical Decision Support, Health Base Foundation, Houten, Netherlands; ^3^Department of Pharmaceutical and Pharmacological Sciences, Unit of Clinical Pharmacology and Pharmacotherapy, Catholic University of Leuven, Leuven, Belgium; ^4^Dutch Medicines Evaluation Board (CBG-MEB), Utrecht, Netherlands; ^5^Department of Clinical Pharmacy and Pharmacology, University of Groningen, University Medical Centre Groningen, Groningen, Netherlands; ^6^Department of Gastroenterology and Hepatology, Erasmus University Medical Centre, Rotterdam, Netherlands; ^7^Department of Hospital Pharmacy, Erasmus University Medical Centre, Rotterdam, Netherlands

**Keywords:** medicines information, Summary of Product Characteristics (SmPC), hepatic impairment, European Medicines Agency (EMA) guideline, prescribing information

## Abstract

**Background:** In 2005, the European Medicines Agency (EMA) released guidance on pharmacokinetic studies in patients with hepatic impairment. This guidance describes the design of these studies and what information should be presented in the Summary of Product Characteristics (SmPC). We aim to evaluate the availability and clinical applicability of information on medicine use in patients with hepatic impairment in SmPCs and registrational dossiers of recently approved medicines.

**Methods:** We reviewed SmPC information on use in patients with hepatic impairment of 51 new medicines authorized between 2015 and 2017. Per medicine, we assessed the availability of nine information items derived from the EMA guidance, i.e. type of hepatic disease studied; stratification by severity of hepatic impairment; influence of hepatic impairment on the pharmacokinetics; safety advice in mild, moderate, and severe hepatic impairments; and dosing recommendation in mild, moderate, and severe hepatic impairments. If unavailable, the European Public Assessment Report (EPAR) and study report were consulted consecutively. Of available items, clinical applicability was assessed by labeling information as “clear” or “ambiguous”.

**Results:** Of 51 medicines, 15 had no pharmacokinetic study in patients with hepatic impairment described in their SmPC. The other 36 SmPCs contained on average seven of the nine information items (range 4–9). One SmPC contained all 9 items, and after consulting, the study reports, 11 SmPCs were complete. The item “type of hepatic disease studied” was available in one SmPC, though it could be retrieved in 21 study reports. Regarding clinical applicability, there was no medicine with all information items available and clearly formulated in the SmPC. A total of 12 medicines (33%) contained only clearly formulated information, while 24 (67%) contained at least one ambiguously formulated information item (range 0–4). Items often ambiguously formulated were: “definition of mild, moderate, and severe hepatic impairment” (15 ambiguous SmPCs) and “safety advice in severe hepatic impairment” (17 ambiguous SmPCs).

**Conclusion:** While SmPCs contain a large part of information requested by the EMA, clinical applicability seems low, as it is often unclear to which specific type of hepatic disease patient the advice applies. This can negatively influence the practical use by healthcare professionals.

## Introduction

Patients with hepatic impairment are at risk for adverse drug reactions when using medicines as drug concentrations could increase due to pharmacokinetic (PK) changes (Delco et al., 2005; Verbeeck, 2008). The influence of hepatic impairment on the PK of a medicine depends on the type and severity of the underlying hepatic disease (Hughes, 2008; Verbeeck, 2008). Cirrhosis, the advanced stage of all chronic liver diseases, has the largest influence on drug concentrations (Ohnhaus et al., 1982; Morgan and McLean, 1995; Lill et al., 2000; Verbeeck, 2008). Research demonstrated that nearly 30% of patients with cirrhosis suffer from adverse drug reactions and that almost 80% of these reactions was possibly preventable because inadequate dosages or contraindicated medicines were used (Franz et al., 2013).

The knowledge on dose adjustments and contraindications for medicines in patients with hepatic impairment is often based on the results of PK studies conducted by pharmaceutical companies. Realizing that information was not always generated to the same extent for different medicines, the European Medicines Agency (EMA) published a guideline on the evaluation of PK in patients with hepatic impairment in 2005 (European Medicines Agency, 2005). This guideline provides recommendations on the design and reporting of PK studies in subjects with impaired hepatic function. The results from these PK studies are presented in a study report and discussed in the European Public Assessment Report (EPAR). In the Summary of Product Characteristics (SmPC), the safety and dosing recommendations resulting from the PK studies are presented to healthcare professionals.

Previous research indicated possible shortcomings in information provided in SmPCs on medication use in patients with hepatic impairment. A small study from 2002 reported that the advice in SmPCs was often inconsistent, unclear, and unhelpful (Anonymous, 2001). A more recent study (2013) evaluated prescribing guidance on patients with hepatic impairment in USA Food and Drug Administration (FDA)-approved labels and reported non-specific dose recommendations (Chang et al., 2013). No previous study has assessed the quality of prescribing information in SmPCs after release of the EMA guideline, nor in other authorization documents. Our aim is therefore to evaluate the availability of specific information on the use of a medicine in patients with hepatic impairment in SmPCs, EPARs, and study reports of recently approved medicines and to evaluate the clinical applicability of the SmPC information.

## Materials and Methods

We included all human medicines authorized through a centralized procedure by the EMA from 2015 until 2017 containing a new chemical entity. The EMA guideline recommends studies in patients with hepatic impairment if medicines are likely to be used in this population, and if hepatic impairment is likely to influence PK (European Medicines Agency, 2005). To focus our analysis on these medicines, we excluded single-use medicines (such as vaccines), non-systemic locally acting medicines, orphan medicines, and medicines that had a conditional approval or were approved under exceptional circumstances. Fixed-dose combination medicines were also excluded since these often contain one advice based on PK alterations and studies of two or more medicines which was difficult to incorporate in our method.

We used data from three different authorization documents: SmPCs, EPARs, and study reports. The SmPCs and EPARs were retrieved from the EMA website (https://www.ema.europa.eu) in April and May 2018. The versions of the documents available on this website correspond to the most recently updated version. Study reports are often not or only partially publicly available, and individual patient data (such as medical histories of study subjects) are also not (yet) published (European Medicines Agency, 2019). If available, study reports were accessed through the EMA’s clinical data website (https://clinicaldata.ema.europa.eu). The non-publicly available study reports and individual patient data were accessed at the Dutch Medicines Evaluation Board.

**Figure 1:**
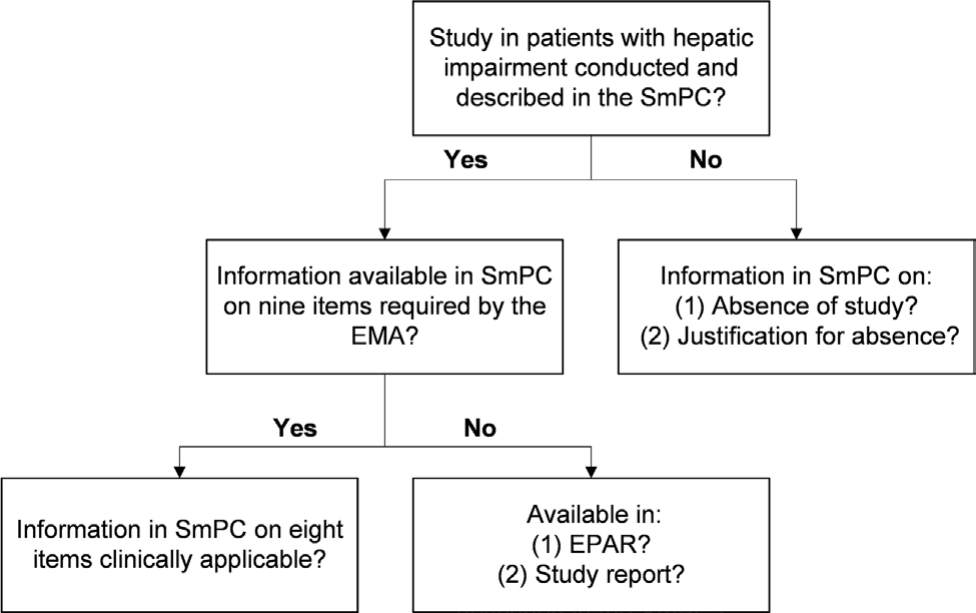
Flowchart of steps followed per medicine. EMA, European Medicines Agency; EPAR, European Public Assessment Report; SmPC, Summary of Product Characteristics.

Per medicine, we examined the SmPC to assess whether a study in patients with hepatic impairment was conducted and described (**Figure 1**). If a study was described in the SmPC, the availability of the information and the clinical applicability were assessed. For medicines without a study in hepatic impairment, we assessed if this was explicitly mentioned in the SmPC and if a justification was given for its absence.

### Assessment of Information Availability and Clinical Applicability

For the assessment of information availability, we used nine information items derived from the section about labeling of the EMA guideline (**Table 1**) (European Medicines Agency, 2005). The guideline describes that characteristics of the patients included in the hepatic impairment study should be stated in the SmPC which we assessed by two information items: (Verbeeck, 2008) description of the type of hepatic disease studied and (Delco et al., 2005) stratification by severity of hepatic impairment. The third information item included a description of the influence of hepatic impairment on the PK. Concerning the remaining information items: the EMA states that specific recommendations should be given on the use (e.g., warnings, precautions) and dosing of medicines in patients with hepatic impairment. We incorporated this into one information item on the safety and one on dosing per severity of hepatic impairment (i.e., mild/moderate/severe) (six information items). A safety and dosing recommendation could be related to the same sentence. For example, the dosing recommendation “no dose adjustment needed” was also counted as safety recommendation since it implies that the medicine can be used. Four of the researchers (RW, LT, MM-S, KT) established the nine information items in consensus.

**Table 1:** Content of section 5 “labeling issues” from the EMA guideline on the evaluation of pharmacokinetics in patients with hepatic impairment (European Medicines Agency, 2005).

“Specific dosing recommendations should be given in section 4.2 with cross-reference to section 5.2, and, when relevant, to sections 4.3 and/or 4.4. The characteristics of the subjects included in the hepatic impairment study should be stated in section 4.2, and extrapolations should not be made beyond what has actually been studied. Efforts should be made to describe the change in pharmacokinetics related to changes in clinical parameters like S-albumin, S-bilirubin, or prothrombin time (preferably expressed in terms of the international normalized ratio, INR) if a relationship has been found. Even when no posology adjustment is needed, this should be stated in section 4.2. Lack of information regarding influence of hepatic impairment on the pharmacokinetics could result in a contraindication or warning, depending on the characteristics of the drug. When precaution is recommended and no specific dose recommendations can be given, measures to be taken by the prescriber (e.g., careful monitoring) should be specified. Information regarding the influence of hepatic impairment on the pharmacokinetics should be given in the special populations subsection of section 5.2, with cross-reference to section 4.2 if posology adjustment is needed and 4.5 if interactions may be changed. The information should include which type of hepatic disease has been studied, effects on parent compound and metabolites and, when relevant, include effects on protein binding and unbound exposure. Also when pharmacokinetics in patients with hepatic impairment has not been evaluated, this information should be given in section 5.2. When relevant, information that hepatic impairment is unlikely to affect the pharmacokinetics to a clinically relevant extent could be included if this has been well justified.” (European Medicines Agency, 2005).

Per medicine, we evaluated if the nine information items were available in the SmPC (**Table 2**). For unavailable items, we first consulted the EPAR to assess if the item was found there and if still not available; the study report was checked. Of the available information items in the SmPC, the clinical applicability was assessed (**Table 2**). Per item, we evaluated the formulation of the SmPC information and labeled it as “clear” or “ambiguous” information. This assessment focused on the applicability of the advice to healthcare professionals: is it clear to which patients the advice applies and what the healthcare professional should do? As the item “the influence of hepatic impairment on the pharmacokinetics” is neither a description of the “at-risk population,” nor an instruction, this item was not evaluated. The clear description of the characteristics of the patients was assessed based on a previous study (Anonymous, 2001). The clinical applicability of the safety and dosing recommendations was based on a study by Salgado *et al*. (Salgado et al., 2013). If there was no clear statement that the medicine can or cannot be used, the safety recommendation could only be labeled as “clear” information if there were safety actions specified for prescribers. This is in line with the EMA guideline: “when precaution is recommended and no specific dose recommendations can be given, measures to be taken by the prescriber (e.g., careful monitoring) should be specified.” (**Table 1**) (European Medicines Agency, 2005).

**Table 2:** Method used for assessing the availability and clinical applicability of information in SmPCs.

No.	Assessment of availability	Assessment of clinical applicability		
	Is the following information item available?	Description	Example sentences	
			Clear information	Ambiguous information
**Characteristics of patients**				
1.	Type of hepatic disease studied	Is the patient group clearly described? Is the term “hepatic impairment” defined?	“Patients with cirrhosis”	“Patients with hepatic impairment”
2.	Stratification by severity of hepatic impairment	Are the terms used to grade the severity of hepatic impairment defined?	“Mild hepatic impairment (Child–Pugh A)”	“Mild hepatic impairment”
**Influence on pharmacokinetics**				
3.	Influence of hepatic impairment on the pharmacokinetics	Clinical applicability not tested		
**Safety recommendations**				
4. 5. 6.	Advice on safety in patients with: Mild hepatic impairment Moderate hepatic impairment Severe hepatic impairment	Clear statement that medicines can or cannot be used or which safety actions are needed^a^	“Contraindicated,” “use with caution while monitoring …, “ “dose adjustment (not) needed”	“Use with caution,” “it is preferable to,” “not recommended to use”
**Dosing recommendations**				
7. 8. 9.	Dosing recommendations in patients with: Mild hepatic impairment Moderate hepatic impairment Severe hepatic impairment	Specified dose adjustment or stating that no dose adjustments are necessary^a^	“Adjust dose to 500 mg once daily,” “no dose adjustment is necessary,” “contraindication”	“Dose adjustment is necessary”

a These items were assessed for every severity class (i.e., mild, moderate, and severe).

### Analyses

Two authors (LT, RW) evaluated the availability and clinical applicability of information in the SmPCs and EPARs and discussed in case of discrepancies. If the authors still disagreed, a discussion was held with two other researchers (MM-S, KT) until consensus was reached. RW examined the study reports together with an employee of the Dutch Medicines Evaluation Board (MM-S). The results were analyzed using Microsoft Excel and reported with descriptive statistics.

## Results

**Figure 2:**
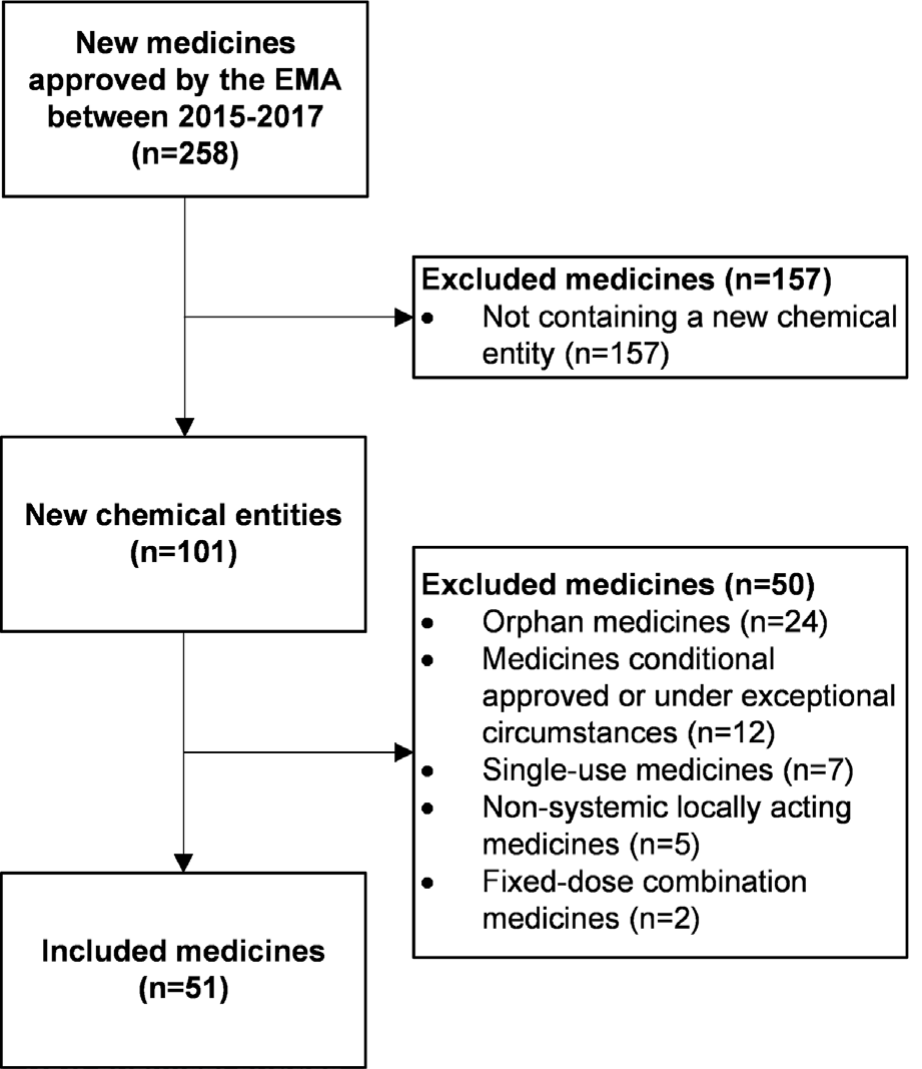
Flowchart of inclusion of medicines. *EMA, European Medicines Agency*.

From 2015 until 2017, the EMA authorized a total of 258 new human medicines, and 101 were new chemical entities (**Figure 2**). We included 51 medicines in our study (**Table 3**). With 27 (53%) of these, a dedicated PK study was conducted in patients with hepatic impairment. For nine medicines (18%), the SmPC described a population PK analysis, and for three of these nine, a dedicated PK study is currently ongoing or recently finished.

**Table 3:** Characteristics of the included medicines (n = 51).

	n	%
**Year of authorization**		
2015	20	39
2016	15	29
2017	16	31
**Therapeutic area**		
Alimentary tract and metabolism	2	4
Blood and blood forming organs	7	14
Cardiovascular system	3	6
Dermatological drugs	1	2
Genitourinary system and reproductive hormones	1	2
Systemic hormonal preparations	1	2
Antiinfectives for systemic use	5	10
Antineoplastic and immunomodulating agents	24	47
Musculoskeletal system	1	2
Nervous system	4	8
Respiratory system	2	4
**Study in patients with hepatic impairment**		
Dedicated pharmacokinetic study	27	53
Population pharmacokinetic analysis	9	18
No study	15	29
**Total**	**51**	**100**

For 15 (29%) included medicines, no study was performed to assess the impact of hepatic impairment on the PK. Thirteen of these were monoclonal antibodies or other medicines not metabolized or eliminated by the liver. The remaining two are partly excreted in feces and have no ongoing dedicated PK study. Eleven of these 15 medicines also described in the SmPC that no study was performed, and eight provided a justification for the absence of a hepatic impairment study.

### Availability of Information

Of the 36 medicines with a PK study, the SmPCs contained on average 7 of the 9 information items (range 4–9). The items “influence of hepatic impairment on the pharmacokinetics,” “safety advice in mild hepatic impairment,” and “dosing recommendation in mild hepatic impairment” were found in the SmPCs of all of these 36 medicines (**Table 4**). Low scoring items were “the type of hepatic disease studied” (n = 1, 3%) and “dosing recommendation in severe hepatic impairment” (n = 16, 44%). The number of medicines with all nine information items available increased from one (3%) after the SmPC and EPAR evaluation, to 11 (31%) medicines after consulting the study report. Of all the study reports consulted (n = 35), 10 (29%) were publicly available on the EMA website.

**Table 4:** Availability of information on patients with hepatic impairment in authorization documents of 36 medicines.

Information item	SmPC		EPAR		Study report	
	n	%	n	%	n	%
Type of hepatic disease studied	1	3	1	3	22	61
Stratification by severity of hepatic impairment	35	97	35	97	35	97
Influence of hepatic impairment on the pharmacokinetics	36	100	36	100	36	100
Safety advice in mild hepatic impairment	36	100	36	100	36	100
Safety advice in moderate hepatic impairment	33	92	33	92	33	92
Safety advice in severe hepatic impairment	31	86	31	86	31	86
Dosing recommendation in mild hepatic impairment	36	100	36	100	36	100
Dosing recommendation in moderate hepatic impairment	28	78	28	78	30	83
Dosing recommendation in severe hepatic impairment	16	44	17	47	17	47

Results are expressed in number and percentage of medicines with the information item available after consulting the SmPC, EPAR, or study report. The additional information from the study reports in terms of type of hepatic disease studied can be found in **Table 5** and the dosing recommendations in moderate hepatic impairment in the published studies of these medicines (Khatri et al., 2015;Gibbs et al., 2017). EPAR, European Public Assessment Report; SmPC, Summary of Product Characteristics.

The SmPC of one medicine described the type of hepatic disease studied which was cirrhosis. This information was available though in 21 study reports. **Table 5** provides an overview of the hepatic diseases documented in these study reports. For five medicines, all patients included in the study had cirrhosis documented in their medical history. For the other medicines, and especially in the mild hepatic impairment group, the medical history of the included patients described a variety of hepatic diseases with and without cirrhosis. In some medical histories, we could not find a (chronic) liver disease documented.

**Table 5:** Overview of hepatic diseases documented in the medical history of the included patients in the study reports of 21 medicines. Expressed in number of patients and stratified by severity of impairment and documentation of cirrhosis.

Documented hepatic disease	Total^a^ (n = 368)		Mild hepatic impairment (n = 115)		Moderate hepatic impairment (n = 166)		Severe hepatic impairment (n = 57)	
	n	%	n	%	n	%	n	%
**Cirrhosis, total**^b^	**264**	**71.7**	**68**	**59.1**	**139**	**83.7**	**43**	**75.4**
Alcoholic liver disease	109	41.3	24	35.3	64	46.0	15	34.9
Viral hepatitis C	139	52.7	39	57.4	72	51.8	20	46.5
Viral hepatitis B	23	8.7	7	10.3	11	7.9	2	4.7
NASH	6	2.3	3	4.4	2	1.4	1	2.3
Other	15	5.7	4	5.9	6	4.3	5	11.6
Unknown	26	9.8	2	2.9	16	11.5	5	11.6
**No cirrhosis documented, total**^b^	**104**	**28.3**	**47**	**40.9**	**27**	**16.3**	**14**	**24.6**
Alcoholic liver disease	24	23.1	10	21.3	8	29.6	2	14.3
Viral hepatitis C	59	56.7	34	72.3	12	44.4	11	78.6
Viral hepatitis B	6	5.8	0	0.0	5	18.5	1	7.1
NASH	8	7.7	4	8.5	3	11.1	1	7.1
Other	17	16.3	5	10.6	6	22.2	1	7.1
Unknown	7	6.7	2	4.3	0	0.0	0	0.0

a The total number of patients contains data from one additional medicine. The medical history of this medicine was not stratified by severity of impairment.

b The individual hepatic diseases do not sum up to the total number because patients could have more than one hepatic disease documented.

NASH, non-alcoholic steatohepatitis.

The SmPCs of 35 (97%) medicines stratified hepatic impairment by severity with 27 describing the use of the Child–Pugh classification (all dedicated PK studies). All of these 27 medicines included patients with moderate hepatic impairment (Child–Pugh B) in their PK study, 25 included patients with mild hepatic impairment (Child–Pugh A), and 14 severe hepatic impairment patients (Child–Pugh C). The remaining eight medicines stratified the severity of hepatic impairment by the National Cancer Institute (NCI) criteria of hepatic dysfunction (all population PK analyses). All eight included patients with NCI mild hepatic impairment in their study, one included patients with NCI moderate hepatic impairment, and none patients with NCI severe hepatic impairment.

**Table 6** gives an overview of the content of the safety and dosing recommendations, stratified by severity of hepatic impairment. Contraindications and dose adjustments were only advised in medicines subjected to a dedicated PK study.

**Table 6:** Overview of safety and dosing recommendations in the SmPCs of 36 medicines, stratified by severity of hepatic impairment.

	Mild hepatic impairment		Moderate hepatic impairment		Severe hepaticimpairment	
	n	%	n	%	n	%
**Safety recommendations**	**36**	**100**	**36**	**100**	**36**	**100**
Can be used (i.e., dose adjustment (not) needed)	31	86	21	58	8	22
Use with caution	4	11	8	22	6	17
Outweigh benefits and risks	0	0	0	0	2	6
Not recommended to use	0	0	3	8	10	28
Should not be used	0	0	0	0	2	6
Contraindication	1	3	1	3	3	8
*None (not available)*	*0*	*0*	*3*	*8*	*5*	*14*
**Dosing recommendations**	**36**	**100**	**36**	**100**	**36**	**100**
Dose adjustment not needed	33	92	19	53	7	19
Dose adjustment needed	2	6	8	22	4	11
Should not be used/contraindication	1	3	1	3	5	14
*None (not available)*	*0*	*0*	*8*	*22*	*20*	*56*

### Clinical Applicability of Information

**Figure 3:**
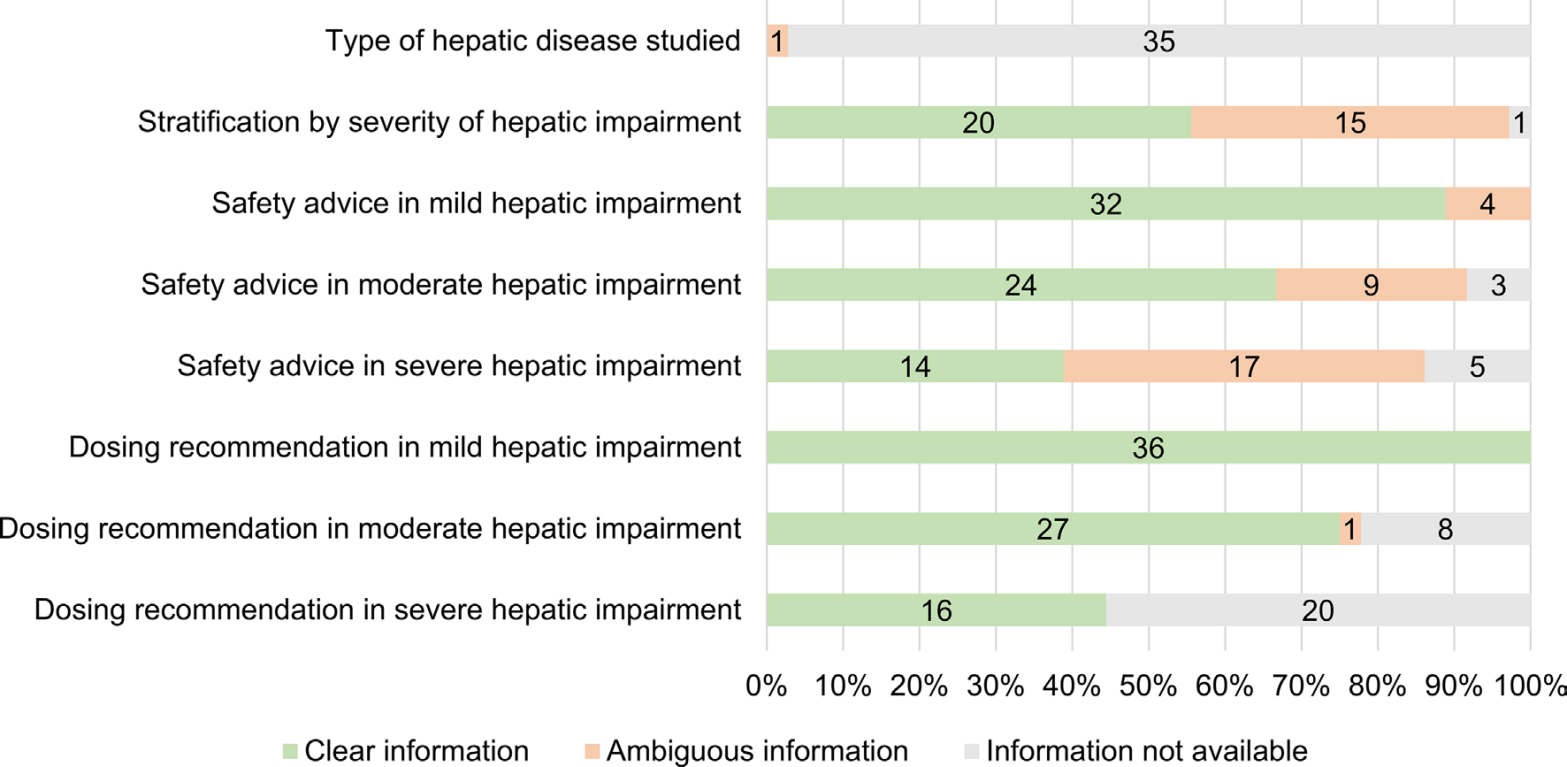
Number and percentage of medicines with clear, ambiguous or unavailable information in SmPCs (n = 36).

**Figure 3** shows the clinical applicability assessment of the SmPC information of the 36 medicines with a PK study. **Table 7** provides examples of clear and ambiguous information in SmPCs. When available, dosing recommendations were almost always formulated clearly, while information on the definition of mild/moderate/severe hepatic impairment (20 clear SmPCs, 56%) and the safety advice in severe hepatic impairment (14 clear SmPCs, 39%) was often ambiguously formulated. The type of hepatic disease studied was only present in one SmPC but ambiguously formulated. Four different wordings were used interchangeably (hepatic impairment, chronic liver disease, pre-existing hepatic impairment, and hepatic cirrhosis) to define the “at-risk population” (**Table 7**). There was no medicine with all information items available and clearly formulated. A total of 12 medicines (33%) contained only clearly formulated information, while 24 (67%) contained at least one ambiguously formulated information item (range 0–4).

**Table 7:** Results of the assessment of the clinical applicability of the information: examples of clear and ambiguous information described in SmPCs concerning the use in patients with hepatic impairment.

Clearly formulated information	Ambiguously formulated information
**Patient characteristics**	
**All terms to grade severity of hepatic impairment are defined**: “Section 4.2: No dose adjustment is required in patients with **mild hepatic impairment (Child–Pugh A)**. The dose should be reduced to 5 mg once daily in patients with **moderate hepatic impairment (Child–Pugh B)** (see sections 4.4 and 5.2). Tofacitinib should not be used in patients with **severe hepatic impairment (Child–Pugh C)** (see section 4.3).” (Pfizer Ltd, 2017)	**Ambiguous information on type of hepatic disease because of all the different terms used, no clear definition:** “Section 4.2: Exposure to brivaracetam was increased in adult patients with **chronic liver disease**. (…) A maximum daily dose of 150 mg administered in two divided doses is recommended for **all stages of hepatic impairment**. (…) Section 4.4: There are limited clinical data on the use of brivaracetam in patients with **pre-existing hepatic impairment**. Dose adjustments are recommended for patients with hepatic impairment. Section 5.2: A pharmacokinetic study in subjects with **hepatic cirrhosis** (Child–Pugh grades A, B, and C) showed similar increases in exposure to brivaracetam irrespective of disease severity (50, 57, and 59%), relative to matched healthy controls.” (UCB Pharma SA, 2016) **Definition lacking of terms to stratify severity of impairment**: “Section 4.2: No dose adjustment is required in patients with **mild or moderate hepatic impairment**. Baricitinib is not recommended for use in patients with **severe hepatic impairment**. Section 5.2: There was no clinically relevant effect on the PK of baricitinib in patients with **mild or moderate hepatic impairment**. The use of baricitinib has not been studied in patients with **severe hepatic impairment**.” (Eli Lilly, 2017)
**Safety advice in patients with mild/moderate/severe hepatic impairment**	
**Caution is explained:** “There are no data in patients with severe hepatic impairment (see section 5.2). Rolapitant should be **used with caution** in these patients. If use cannot be avoided, patients should be **monitored for adverse reactions** to rolapitant (see section 4.8).” (Tesaro UK Ltd, 2017)	**Ambiguous safety advice in moderate and severe hepatic impairment:** “No dose adjustment of dasabuvir is required in patients with mild hepatic impairment (Child–Pugh A). Dasabuvir **is not recommended in** patients with moderate hepatic impairment (Child–Pugh B) (see sections 4.4 and 4.8). Dasabuvir **should not be used** in patients with severe hepatic impairment (Child–Pugh C) (see section 5.2).” (AbbVie Ltd, 2015)
**Dosing recommendation in patients with mild/moderate/severe hepatic impairment**	
**Dosing advice specified**: “Section 4.2: No dose adjustment of palbociclib is required for patients with mild or moderate hepatic impairment (Child–Pugh classes A and B). For patients with severe hepatic impairment (Child–Pugh class C), **the recommended dose** of palbociclib **is 75 mg once daily** on schedule 3/1 (section 4.4, 5.2)” (Pfizer Ltd, 2016)	**Concrete dose recommendation in moderate hepatic impairment lacking:** “Section 4.2: No dose adjustment is necessary in patients with mild hepatic impairment (Child–Pugh Class A). There is limited clinical experience in patients with moderate hepatic impairment (Child–Pugh Class B). Caution must be exercised in these patients and **dose adjustment may be necessary** (see section 5.2). There is no clinical experience in patients with severe hepatic impairment (Child–Pugh Class C); therefore, opicapone is not recommended in these patients (see section 5.2).” (Bial - Portela and Ca SA, 2016)

For clarity, brand names have been replaced by generic medicines names in all examples.

## Discussion

In this study, we reviewed SmPC information on patients with hepatic impairment of 51 recently approved medicines and found that 36 described a PK study in patients with hepatic impairment in their SmPC. On average, 7 of 9 information items requested by the EMA were available in these SmPCs. Yet, safety advice or dose recommendations for patients with severe hepatic impairment were unavailable for almost 60% of evaluated medicines and/or ambiguously formulated. Essential information on the type of hepatic disease of patients included in the required PK studies was lacking for 35 of 36 medicines but could be retrieved for 21 medicines in the non-publicly available part of the study report. Based on the documentation in the study reports, we could not confirm that the appropriate patients were studied in all PK studies. In addition, in more than 40% of evaluated medicines, the severity of hepatic impairment of the studied patients was not clearly specified in the SmPC.

A substantial part of information requested in the EMA guideline was available in the SmPCs of the medicines in our sample that conducted a PK study. For the other medicines, the lack of a hepatic impairment study was often justified by negligible hepatic clearance of the particular medicinal product which is accepted by the EMA (European Medicines Agency, 2005). Two earlier studies found different results. A study from 2001 showed that only a few of the 25 studied SmPCs gave specific, detailed advice on the use of a medicine in patients with hepatic impairment (Anonymous, 2001). Chang and colleagues (Chang et al., 2013) observed that a large part of FDA labels provided dosing recommendations, but these recommendations were in 60% of labels not stratified by severity of hepatic impairment while almost all SmPCs in our sample did so. This may be explained by different requirements between regulatory agencies, and differences between US and EU labelings on hepatic impairment have been previously noted (Bjornsson et al., 2015). Considering though that applicants usually conduct a single PK study in patients with hepatic impairment that is submitted to all regulatory authorities, it is more likely that the enhanced information in SmPCs is explained by an improvement in the quality of information over time, as these earlier included medicines were approved before March 2001, respectively 2011.

Prescribing information about patients with severe hepatic impairment was often lacking or ambiguously formulated. This was probably caused by a lack of clinical data: in the PK study of only 14 medicines, patients with severe hepatic impairment were included. Previous studies also showed that, with increasing severity of hepatic impairment, less prescribing information is available (Periáñez-Párraga et al., 2012;Chang et al., 2013). If information on patients with severe hepatic impairment was available, it was frequently ambiguously formulated. Vague statements such as “not recommended to use” leave it open for interpretation whether the medicine is absolutely contraindicated and what would be the circumstances of usage. Ambiguous formulations such as “use with caution,” “not recommended to use,” and “should not be used” were also observed in studies examining SmPC recommendations in other clinical areas such as renal impairment (Geerts et al., 2012; Beers et al., 2013; Salgado et al., 2013; Arguello et al., 2015). This finding should be seen in the light of the ethical and practical difficulties faced with research in such a vulnerable patient group. Yet, although no clinical data are available, measures to be taken by the prescriber could still be specified or explained in the SmPC (see **Table 7**, example with rolapitant) as also advised by the EMA (European Medicines Agency, 2005).

Another important finding was that the type of hepatic disease of patients included in PK studies was not specified in the SmPC text, even though specifically requested by the EMA guideline (European Medicines Agency, 2005). As shown in literature, prescribers other than gastroenterologists often do not know which patients with a liver disease need dose adjustments or avoidance of certain medicines (Rossi et al., 2008; Nguyen et al., 2014) that is possibly caused by the use of the undefined term “hepatic impairment.” In the one medicine where the type of hepatic disease studied was available in the SmPC, the recommendations were ambiguous because different wordings were used interchangeably to define the “at-risk population.” The study from 2001 already concluded that this “at-risk population” was often vaguely described (Anonymous, 2001), so it seems little to no progress has been made in this area. We could find the information on the hepatic disease that caused the impairment for most medicines in the study reports. But contrary to SmPCs and EPARs, most of these reports are not (yet) accessible to healthcare professionals. The EMA is trying to increase transparency by providing access to clinical study data on a website (European Medicines Agency, 2019); yet, we noticed we could only find study reports for 29% of the medicines. More importantly, it appears that regulators are not aware that the hepatic disease information is relevant to the healthcare professionals. We recommend to include this information in the SmPCs.

The FDA label study (Chang et al., 2013) described the explicit use of standardized terminology such as the Child–Pugh score as solution for the non-specific phrase “hepatic impairment.” In our sample, all dedicated PK studies used this score. An important remark to the Child–Pugh classification is that it was not intended and validated as a measure to assess the remaining capacity of the liver to eliminate medicines (European Medicines Agency, 2005). As the guideline also recommends, appropriate use of the Child–Pugh classification is important because the parameters are not specific of hepatic (elimination) impairment (Durand and Valla, 2005;European Medicines Agency, 2005). For example, everyone scores 5 points (i.e., class A: “mild hepatic impairment”) because that is the minimum score and increases in one of the parameters due to other causes (e.g., a bilirubin increase due to hemolysis or a prolonged INR due to coumarin use) could even result in a “moderate hepatic impairment” classification. The guideline provides no further details on appropriate use; however, in clinical practice, the Child–Pugh score is intended to assess the severity and prognosis of cirrhosis (Pugh et al., 1973; Durand and Valla, 2005). Prior literature also concluded that hepatic elimination was not significantly impaired in a variety of chronic liver diseases unless cirrhosis was present (Morgan and McLean, 1995; Verbeeck, 2008). Based on the data we found in the study reports, we cannot confirm that the Child–Pugh classification was used appropriately in all studies. Most of the subjects in the PK studies had documented cirrhosis, and the Child–Pugh classification was used as intended. In the remaining subjects, insufficient details were provided to assess if the hepatic elimination capacity was relevantly impaired because there was no cirrhosis documented and not even a (chronic) liver disease for some. Inappropriate use of the Child–Pugh classification in clinical studies may result in an underestimation of the changes in PK in patients with hepatic impairment due to cirrhosis. Because of its limitations, in further research, alternatives for the Child–Pugh classification should be explored.

### Strengths and Limitations

We performed an in-depth analysis of the hepatic impairment information in authorization documents. Unique to our study is the access we had to the non-publicly available part of the study reports. We studied a limited number of medicines, making comparisons between therapeutic groups or between SmPC information over time not possible. Hence, our results cannot be generalized to older medicines, especially not those authorized before publication of the guideline in 2005. Furthermore, we only studied medicines that were granted market authorization *via* a centralized procedure, so results are not necessarily valid for medicines authorized through a national or decentralized procedure. Nevertheless, in national and decentralized procedures, use of the EMA guidelines is also recommended.

### Implications

The lack of clear guidance in SmPCs on patients with severe hepatic impairment can be challenging for healthcare professionals treating these severely ill patients who need medicines but are very sensitive to PK and pharmacodynamic alterations. As there are practical and ethical issues involved in conducting pre-registration studies in patients with severe hepatic impairment, it would be helpful to collect post-marketing data. Further research could explore the potential of registries as information source on treatment and outcome in that patient group.

The EMA reinforced in their hepatic impairment guideline the need for further research to strengthen and improve the guideline (European Medicines Agency, 2005). We recommend to update the guideline on three points. First, the guideline must mention that all terms used to describe the severity of hepatic impairment in the SmPC should also be defined [e.g., patients with mild hepatic impairment (Child–Pugh A)]. Although these definitions are easy to include, more than 40% of SmPCs did not provide this information. Second, the guideline describes that if precautious use of a medicine is advised, SmPCs should also specify measures to be taken by the prescriber (European Medicines Agency, 2005). Nevertheless, we noticed a high prevalence of ambiguous safety advice that lacked such specifications. Therefore, this should be better expressed in the guideline and perhaps also better monitored by the regulators. Finally, we showed that the main weakness of the guideline is the vague term “hepatic impairment” that leaves room for interpretation. Pharmaceutical companies and regulators interpret this differently resulting in a diversity of patient populations in the PK studies. Healthcare professionals as well can have difficulties to interpret “hepatic impairment,” possibly resulting in non-optimal advice, under- or overdosing. As there is no generally accepted definition for the term “hepatic impairment,” its use is not helpful in clinical practice (Bjornsson et al., 2015). Therefore, the EMA guideline needs to be updated to include a more precise definition. Perhaps, it is even better not to use the ambiguous term “hepatic impairment” anymore. Instead, we recommend to use the clearly defined term “liver cirrhosis” in authorization documents, but also in online drug reference works and in the published PK studies. These activities may prevent prescribing problems in practice, such as the use of inadequate dosages or contraindicated drugs in patients with cirrhosis, as demonstrated by Franz *et al.* (Franz et al., 2013). In the Netherlands, the drug-disease interaction “hepatic impairment” has been replaced in clinical decision support systems by a new drug-disease interaction “liver cirrhosis” to better support healthcare professionals (Weersink et al., 2016; Weersink et al., 2018).

## Conclusion

In this study, we have shown that SmPCs of recently approved medicines contain a large part of the information required by the EMA guideline on patients with hepatic impairment. Although available, the safety advice was often ambiguously formulated and therefore not *per se* clinically applicable. Unclear advice on patients with severe hepatic impairment was often explained by a lack of research. Information on the type of hepatic disease was often lacking in the SmPC but could be found in the non-publicly available part of the study report. We recommend that such information should be included in SmPCs. This information is also needed to judge if the Child–Pugh classification was used appropriately, because the parameters it includes are not specific of hepatic (elimination) impairment. Based on our results, we cannot conclude that the appropriate patients were studied in all hepatic impairment studies. We specifically recommend to update the 2005 EMA guideline to use the clearly defined term “liver cirrhosis” instead of “hepatic impairment”. This will support pharmaceutical companies in conducting and reporting PK studies in the most relevant patients with hepatic disease and healthcare professionals when prescribing for these vulnerable patients.

## Data Availability

The dataset used in these analyses are available upon request to interested researchers. The SmPCs and EPARs assessed in this study are publicly available on the website of the EMA (https://www.ema.europa.eu). The evaluated clinical study reports are not publicly available and cannot be supplied because of confidentiality. Requests to access these reports should be directed to the Marketing Authorization Holder.

## Author Contributions

RW, LT, MM-S and KT participated in data analysis and interpretation. RW and LT drafted the manuscript. MM-S, KT, PM, HM and SB critically revised the manuscript. Supervision was done by KT and MM-S. All authors approved the final version of the manuscript.

## Funding

This study was funded by the Dutch Medicines Evaluation Board (CBG-MEB).

## Conflict of Interest Statement

MM-S and PM are employed by the Dutch Medicines Evaluation Board (CBG-MEB).

The authors declare that the research was conducted in the absence of any commercial or financial relationships that could be construed as a potential conflict of interest.
